# Effect of the replacement of dietary vegetable oils with a low dose of extravirgin olive oil in the Mediterranean Diet on cognitive functions in the elderly

**DOI:** 10.1186/s12967-018-1386-x

**Published:** 2018-01-19

**Authors:** Elisa Mazza, Antonietta Fava, Yvelise Ferro, Stefania Rotundo, Stefano Romeo, Domenico Bosco, Arturo Pujia, Tiziana Montalcini

**Affiliations:** 1 Department of Medical and Surgical Science, Nutrition Unit, University Magna Grecia, Catanzaro, Italy; 2 Department of Molecular and Clinical Medicine, University of Gothenburg, Gothenburg, Sweden; 3 Neurology Unit, S. Giovanni di Dio Hospital, Crotone, Italy; 4 Department of Clinical and Experimental Medicine, Nutrition Unit, University Magna Grecia, Viale S. Venuta, 88100 Catanzaro, Italy

**Keywords:** Cognitive functions, Elderly, Mediterranean Diet, Extravirgin olive oil

## Abstract

**Background:**

Numerous studies have investigated the role of the monounsaturated fatty acid and other dietary factors in the prevention of cognitive decline but the short-term effect of a low dose of extravirgin olive oil on cognitive performances in the elderly have not still been investigated. Our aim was to investigate whether the replacement of all vegetable oils with a lower amount of extravirgin olive oil, in the contest of a Mediterranean Diet, would improve cognitive performances, among elderly Italian individuals.

**Methods:**

180 elderly individuals were randomly assigned to these treatment groups for 1 year: (1) MedDiet plus extravirgin OO, 20–30 g/day; (2) control MedDiet. The cognitive sub-test of ADAScale was used to detect cognitive decline progression over 12 months.

**Results:**

ADAS-cog score variation after 1 year, adjusted for food groups which were different between groups, was − 1.6 ± 0.4 and − 3.0 ± 0.4 in the MedDiet and MedDiet plus extravirgin OO groups, respectively (p = 0.024). Extravirgin OO intake was 30 g ± 12 and 26 g ± 6 in the MedDiet and MedDiet plus extravirgin OO groups, respectively (p = 0.044).

**Conclusions:**

We demonstrated the higher short-term improvement of cognitive functions scores in individuals of the MedDiet plus low dose of extravirgin olive oil rather than MedDiet alone. Extravirgin olive oil is the best quality oil and may have a neuroprotective effect.

**Electronic supplementary material:**

The online version of this article (10.1186/s12967-018-1386-x) contains supplementary material, which is available to authorized users.

## Background

Individuals with cognitive impairments and dementia increase in prevalence exponentially with age, with trends worldwide likely to worsen in ensuing decades [1]. These clinical conditions are associated with high overall costs representing a severe burden to society which need to plan wisely to allocate appropriate resources to meet the demands of the disease. Thus, all non-pharmacologic measures are also important. Several observational studies and randomized controlled trials have shown the protective role of the Mediterranean Diet (MeDiet) against cognitive decline and in decreasing the risk of developing Alzheimer disease (AD) [2–7].

MeDiet can improve cognitive performances in the elderly [2] and the beneficial effects have been attributed to its high monounsaturated fatty acid (MUFA) content [6] which makes MedDiet a healthy dietary pattern regardless of its high fat content [8]. However, the main concern in the elderly is the change in healthy dietary habits, a reduced interest in food intake and calorie counting, especially in individuals with some degree of cognitive decline [9].

A research suggests that food quality, but not quantity, is a key determinant in order to achieve and maintain a healthy status [10]. Furthermore, there are uncertainties on the notion that increasing MUFA intake per se is effective in improving cognitive functions [11]. In this study, we hypothesized that a change in the quality of vegetable oils would improve the cognitive performances in the elderly better than the quantity. A specific associations between extravirgin olive oil (OO) consumption, and cognitive functions have not still been formally examined, especially at low doses and in the short term.

Thus, our aim was to investigate whether the replacement of all vegetable oils with a lower amount of extravirgin OO, in the contest of a MedDiet, would improve cognitive performances, in the short-term, better than a control MedDiet in a population of elderly individuals.

## Methods

This study included participants recruited into the study entitled: Effect of the MedDiet on cognitive function in the elderly” carried-out between February 2013 to August 2016, funded by Italian Ministry of Health and whose protocol was approved by the local ethics committee at the “Mater Domini” University Hospital in Catanzaro, Italy (projects codes 2011.48).

The participants were from a Mediterranean area (Calabria region, southern Italy) and were invited to participate in the study by newspapers advertisements. All subjects were white, community-dwelling individuals aged ≥ 65 years and had an MMSE score greater than 20 [12]. Participants were literate and were not suffering from any debilitating diseases (like stage 2–5 chronic kidney disease, end stage liver failure, cancer, congestive heart failure) as ascertained from their medical history, a physical and neurological examination and laboratory tests. They had no previous history of cardiovascular disease (CVD) or thyroid dysfunction or excessive alcohol consumption and did not take any dietary supplements, psychotropic drugs. All participants underwent a neuropsychological assessment conducted by an expert neurologist using a medical assessment and the following neuropsychological tests: the Mini Mental State Examination (MMSE) [13–16] and the Alzheimer’s Disease Assessment Scale-Cognitive sub-scale (ADAS-cog) [17–19].

**Fig. 1:**
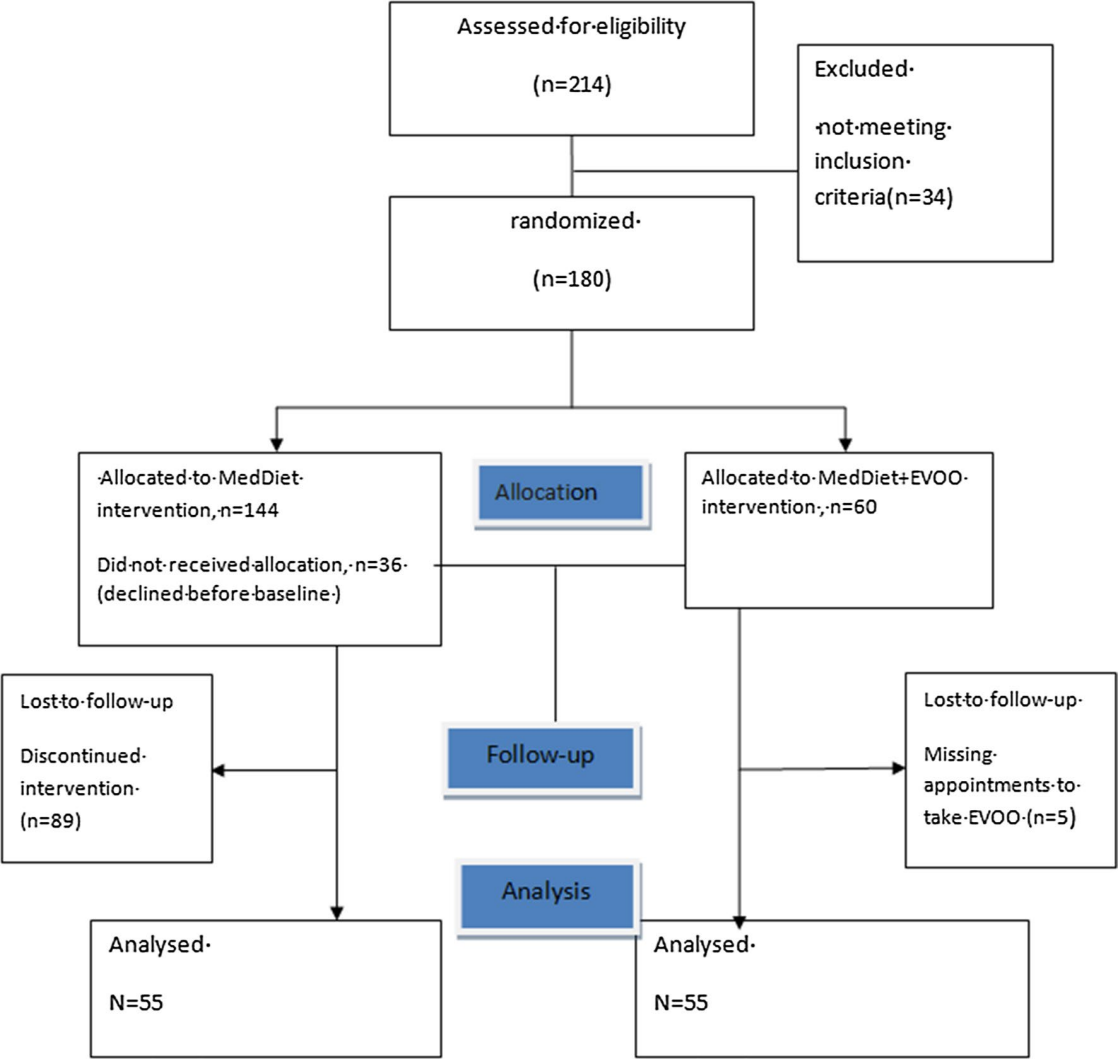
CONSORT study flow-chart

Then 180 participants were randomly assigned to one of the two dietary treatment groups for 1 year (allocation ratio 1:2): (1) a MedDiet in which all vegetable oils (including olive oil, high-oleic safflower oil, high-oleic sunflower oil, canola oil and hydrogenated vegetable oils) were substituted by extravirgin OO at dose of 20–30 g per day, (2) a control MedDiet alone (Fig. 1).

To improve adherence, in the MedDiet plus extravirgin OO group, extravirgin OO was given for free to participants and provided every 3 months by local oils producers (5 L of extravirgin OO for 3 months by Opipari and Torchia Companies, Calabria; extravirgin OO main characteristics: acidity < 0.8%, polyphenol content 280 ppm).

Participants received intensive oral and written recommendations to increase adherence to the MedDiet, reduce all types of fat but energy restriction was not advised for any of the intervention groups. We performed a longitudinal evaluation lasting 12 months with the ADAS-Cog as the main outcome [18].

The investigation conforms to the principles outlined in the Declaration of Helsinki [20]. Written informed consent was obtained from participants.

### Neuropsychological assessment

The neuropsychological assessments were conducted alongside the medical visit and the use of the MMSE and ADAS-cog. MMSE is a global test of cognitive function with components of orientation, attention, calculation, language and recall [13]. A score of 20 or below is indicative of cognitive impairment. A validated Italian version was used [16]. The ADAS-cog is a psychometric scale, measuring memory disturbances, language, praxis, attention and other cognitive abilities [17]. The range of scores is from 0 to 70 and the scale of the ADAS-cog is reversed, where 0 represents no errors and 70 represents errors on all items [18, 19].

Furthermore, we used validated scales to assess behavioural and psychological symptoms and the eventual reduced everyday functional ability which can frequently accompany cognitive decline, such as: Beck Depression Inventory-II (BDI-II), Verbal Fluency (VF), Activities of Daily Living (ADL) and Instrumental Activities of Daily Living (IADL) scales [21–27].

To reduce the potential for practice effects during subsequent visits, different word lists in the neuropsychological tests were used. In addition, the investigators performing the cognitive tests were blinded to the patients’ clinical data and randomization.

### Anthropometric measurements and cardiovascular risk factors assessment

Body weight was measured before breakfast after a 12 h overnight fast with the subjects lightly dressed, subtracting the weight of clothes. Body weight was measured on a calibrated digital scale (model Tanita BC-418MA) accurate to 0.1 kg, and standing height was measured with a wall-mounted stadiometer [28]. BMI was calculated with the following equation: weight (kg)/height (m)^2^. Obesity was defined by the presence of a body mass index (BMI) ≥ 30 kg/m^2^. Waist circumferences and hip circumferences (WC and HC) were measured with a nonstretchable tape over the unclothed abdomen at the narrowest point between costal margin and iliac crest and over light clothing at the level of the widest diameter around the buttocks, respectively, as described in the past [28].

We assessed the presence of the classical cardiovascular (CV) risk factors, such as hyperlipidemia, hypertension, diabetes and smoking, from clinical records and patient interview [12, 28]. Blood pressure was determined at the time of the two visits.

### Dietary intake and cardiovascular risk factors assessment

Dietary intake data were assessed by a 24-h recall and a 7-day food record [12, 29], and calculated using nutritional software MetaDieta 3.0.1 (Metedasrl, San Benedetto del Tronto, Italy). Precisely, the 24-h recall was performed via a face-to-face interview with a dietitian who used images associated with a comprehensive food list. The recall required 15–20 min to complete for each participant. The patients were also asked to report any ingredients, food and food waste in a food diary for a 7-day period. Each patient was trained by a skilled dietitian before starting the study. The dietitian showed how various foods should be recorded. The portion sizes used were based on the typical or natural portion consumed (e.g., a slice of bread, one egg). When a typical portion size was not obvious, a commonly-used portion size was selected (e.g., one cup). The nutrient database used to calculate nutrient intake was derived primarily from INRAN (National Institute of Food Research) 2000 and IEO (European Institute of Oncology) 2008 [12, 29]. This database includes over 5000 foods and brand name products, and is updated annually. Briefly, dietary intake data were entered directly into the software MetaDieta. The software searches for foods and brand products by name. The coding of foods and their variable ingredients occurs as data are entered, with the simultaneous immediate calculation of nutrients. Nutrient values and other food components were generated from the database together with food group assignments. The adherence to MedDiet was assessed at enrollment using the Mediterranean Diet Score (MDS) [4]. Total score ranges from 0 (minimum adherence) to 55 (maximum adherence). A score from 25 to 55 indicates a moderate-high adherence to MedDiet. Furthermore, we assessed the Mediterranean Adequacy Index (MAI) at enrollment and after 1 year [30]. Three levels of adherence were considered: ≤ 2 low score; ≥ 4 high score and 2 ≤ MAI ≤ 4 intermediate score.

### Biochemical evaluation

Venous blood was collected after fasting overnight into vacutainer tubes (Becton & Dickinson, Plymouth, England) and centrifuged within 4 h. Serum glucose, total cholesterol, high density lipoprotein (HDL)-cholesterol, triglycerides and creatinine were measured with Enzymatic colorimetric test. Low-density lipoprotein (LDL) cholesterol level was calculated by the Friedewald formula [31]. Quality control was assessed daily for all determinations.

## Data analysis

Data are reported as mean ± SD. Since an effect size of ADAS-Cog change (ES = mean ADAS-cog difference/baseline SD) of 0.8 has been considered as a large clinically relevant change [32], with 80% power on a two-sided level of significance, a minimum of 25 subjects for each group are required.

A Chi square test was performed to analyze the difference in prevalence between groups and an independent unpaired samples t test was used to compare the means of the two groups at baseline and after 1 year. Changes in MMSE and ADAS cog score from baseline to follow-up (within group variation) were compared using paired Student’s *t* test (two tailed). The general linear model (GLM) was used to adjust the ADAS-cog variation for all variables significantly different between groups at baseline and after 1 year.

Significant differences were assumed to be present at *p* < 0.05 (two-tailed). All comparisons were performed using SPSS 17.0 for Windows (IBM Corporation, New York, NY, United States).

## Results

A total of 110 individuals completed the study (55 participants/group, Fig. 1). Table 1 shows the baseline characteristics of the study population according with the randomization. The two groups were comparable for age, gender, BMI and calories intake. The mean age was 70 ± 4 years, the MMSE score was 24 ± 1 in both groups (p = 0.63 and 0.72 respectively) and ADAS-cog score was 14 ± 4 and 15 ± 5 in the MedDiet and MedDiet plus extravirgin OO groups, respectively (p = 0.14). Furthermore, all other baseline characteristics were not significantly different between groups.

**Table 1:** Baseline participant’s demographic, anthropometric and clinical characteristics according to randomization

Variables	MeDiet (N = 55)	MeDiet plus EVOO (N = 55)	*p* value
Age (years)	70 (4)	70 (4)	0.63
Weight (Kg)	71 (11)	72 (14)	0.68
BMI (Kg/m^2^)	28.0 (5)	28.8 (4)	0.35
WC (cm)	97 (11)	96 (13)	0.97
SBP (mmHg)	128 (15)	132 (11)	0.16
DBP (mmHg)	78 (8)	80 (9)	0.27
Glucose (mg/dL)	104 (34)	101 (21)	0.60
Creatinine (mg/dL)	0.86 (0.2)	0.81 (0.2)	0.21
Total cholesterol (mg/dL)	200 (44)	194 (40)	0.46
HDL cholesterol (mg/dL)	54 (15)	59 (15)	0.14
LDL cholesterol (mg/dL)	128 (41)	124 (34)	0.53
Triglycerides (mg/dL)	123 (61)	108 (55)	0.18
Neuropsychological assessment			
Education level (years)	11 (5)	11 (5)	0.56
MMSE	24.6 (1.3)	24.5 (1.5)	0.72
ADAS-Cog	14.0 (4.5)	15.3 (5.2)	0.14
ADL	6.0 (0.1)	6.0 (0.1)	0.43
IADL	8.0 (0.1)	8.0 (0.2)	0.15
VF	25 (6)	23 (7)	0.15
BDI-III	11 (7)	13 (8)	0.30
Prevalence			
Smokers (%)	52	36	0.36
Hyperlipidemia (%)	48	55	0.56
Lipid-lowering agents (%)	45	50	0.50
Hypertension (%)	52	51	0.90
Antihypertensive agents (%)	50	49	0.60
Diabetes/carbohydrate intolerance (%)	54	41	0.34
Oral hypoglycemic agents (%)	51	40	0.40

*EVOO* extra virgin olive oil, *BMI* body mass index, *WC* waist circumference, *SBP* systolic blood pressure, *DBP* diastolic blood pressure, *HDL* high density lipoprotein, *LDL* low density lipoprotein, *MMSE* mini mental state examination, *ADAS-Cog* Alzheimer’s disease assessment scale-cognitive sub-scale, *ADL* activities of daily living, *IADL* instrumental activities of daily living, *VF* verbal fluency, *BDI-III* Beck depression inventory

The baseline participants’ nutritional intake is showed in Table 2. Energy intake was not significantly different between groups. Only alcohol and cheese consumption were significantly different between groups (alcohol: *9* *g* ± *12* and *5* *g* ± *8* in the MedDiet and MedDiet plus extravirgin OO groups, respectively, p = 0.037; cheese: 48 g ± 39 and 66 g ± 50 in the MedDiet and MedDiet plus extravirgin OO groups, respectively p = 0.038 respectively).

**Table 2:** Nutrients and food groups assessment according to extra virgin olive oil intake-baseline

Nutrients intake	Without EVOO (N = 55)	With EVOO (N = 55)	*p* value
Calories (Kcal)	1880 (452)	1814 (376)	0.40
MAI	2.92 (1)	2.83 (1)	0.67
MDS	33 (3)	33 (3)	0.15
Carbohydrates (%)	46 (6)	47 (7)	0.49
Proteins (%)	17 (2)	17 (3)	0.54
Fats (%)	37 (6)	37 (7)	0.70
Carbohydrates (g)	209 (59)	207 (49)	0.81
Proteins (g)	78 (20)	75 (23)	0.55
Fats (g)	75 (20)	73 (23)	0.63
Monounsaturated fatty acids (g)	38 (9)	37 (12)	0.64
Alcohol (g)	9 (12)	5 (8)	0.037
Food groups intake			
Potatoes (g)	16 (19)	20 (25)	0.30
Cereals (g)	205 (88)	200 (82)	0.77
Legumes (g)	15 (13)	25 (31)	0.051
Vegetables (g)	250 (133)	290 (136)	0.11
Fruit (g)	333 (162)	345 (206)	0.73
Fish (g)	61 (45)	73 (64)	0.27
Meat (g)	79 (44)	75 (48)	0.66
Eggs (g)	11 (10)	15 (28)	0.30
Milk (g)	139 (112)	122 (108)	0.43
Cheese (g)	48 (39)	66 (50)	0.038
Animal fats/margarines (g)	0.69 (2)	1.5 (5)	0.29
Cookies (g)	7.7 (16)	9.6 (16)	0.53
Cakes/pies (g)	28 (24)	38 (30)	0.059
Sugar drinks (g)	27 (89)	10 (30)	0.18
Wine (g)	90 (127)	70 (117)	0.40
EVOO (g)	33 (10)	36 (12)	0.20

*EVOO* extra virgin olive oil, *MDS* Mediterranean Diet Score, *MAI* Mediterranean adequacy index

### Characteristics of the study population and neuropsychological score after 1 year

Clinical characteristics and dietary intake were not significantly different between groups after 1 year, except for extravirgin OO (30 g ± 12 and 26 g ± 6 in the MedDiet and MedDiet plus extravirgin OO groups, respectively, p = 0.044), and carbohydrates, fruit and milk consumption (Table 3).

**Table 3:** Participant’s demographic, anthropometric and clinical characteristics after 1 year according to extra virgin olive oil-intake

Demographic, anthropometric and clinical characteristics	MeDiet (N = 55)	MeDiet plus EVOO (N = 55)	*p* value
Weight (Kg)	71 (11)	71 (14)	0.99
BMI (Kg/m^2^)	27.9 (4)	28.7 (4)	0.29
WC (cm)	96 (11)	95 (12)	0.80
SBP (mmHg)	128 (12)	131 (15)	0.40
DBP (mmHg)	80 (8)	78 (10)	0.34
Glucose (mg/dL)	103 (23)	103 (20)	0.92
Creatinine (mg/dL)	0.86 (0.2)	0.85 (0.2)	0.81
Total Cholesterol (mg/dL)	192 (38)	191 (36)	0.81
HDL Cholesterol (mg/dL)	54 (14)	60 (17)	0.05
LDL Cholesterol (mg/dL)	119 (37)	117 (33)	0.74
Triglycerides (mg/dL)	121 (57)	104 (38)	0.08
Nutrients intake			
Calories (Kcal)	1545 (386)	1560 (292)	0.81
MAI	3.09 (1)	3.22 (1)	0.60
ΔMAI	0.02 (1)	0.18 (1)	0.51
Carbohydrates (%)	47 (5)	51 (6)	0.002
Proteins (%)	16 (3)	17 (3)	0.48
Fats (%)	36 (6)	33 (5)	< 0.001
Carbohydrates (g)	177 (48)	193 (42)	0.053
Proteins (g)	61 (18)	63 (16)	0.43
Fats (g)	60 (17)	55 (12)	0.07
Alcohol (g)	8 (11)	6 (8)	0.21
Monounsaturated fatty acids (g)	31 (10)	28 (5)	0.025
Oleic acid (g)	30 (9)	27 (5)	0.027
Food groups intake			
Potatoes (g)	25 (24)	26 (32)	0.73
Cereals (g)	164 (70)	171 (55)	0.56
Legumes (g)	19 (19)	26 (25)	0.14
Vegetables (g)	203 (295)	229 (128)	0.55
Fruit (g)	269 (149)	336 (188)	0.041
Fish (g)	42 (32)	47 (40)	0.48
Meat (g)	72 (42)	68 (45)	0.64
Eggs (g)	10 (11)	9 (11)	0.87
Milk (g)	104 (98)	145 (114)	0.043
Cheese (g)	35 (29)	35 (26)	0.98
Animal fats/margarines (g)	0.8 (3)	0.7 (3)	0.78
Cookies (g)	8 (16)	12 (17)	0.28
Cakes/pies (g)	25 (18)	20 (20)	0.19
Sugar drinks (g)	4.0 (12)	16 (53)	0.11
Wine (g)	74 (109)	51 (78)	0.20
EVOO (g)	30 (12)	26 (6)	0.044

*EVOO* extra virgin olive oil, *BMI* body mass index, *WC* waist circumference, *SBP* systolic blood pressure, *DBP* diastolic blood pressure, *HDL* high density lipoprotein, *LDL* low density lipoprotein

ADAS-cog scores improved at the second time point in the participants taking extravirgin OO as well as in participants in the MedDiet group (Table 4), but ADAS-cog change was significantly different between groups (ADAS-cog change of − 1.6 ± 2 and − 3.0 ± 3 in the MedDiet and MedDiet plus extravirgin OO groups, respectively, p = 0.018; Table 5). In particular, ADAS-cog score variation, adjusted for alcohol and cheese (baseline intake) and carbohydrates, fruit and milk (intake after 1 year) was − 1.6 ± 0.4 and − 3.0 ± 0.4 in the MedDiet and MedDiet plus extravirgin OO groups, respectively, p = 0.024, Table 5). All other neuropsychological scores were not significantly different between groups after 1 year.

**Tables 4:** Participant’s neuropsychological characteristics after 1 year according to extra virgin olive oil-intake—(within group variation)

Variables	Without EVOO				With EVOO			
	Basal	Follow-up	Δ	*p* value*	Basal	Follow-up	Δ	*p* value*
MMSE	24.6 (1.3)	25.6 (1.8)	0.96 (1.1)	< 0.001	24.5 (1.5)	25.9 (1.3)	1.3 (1.1)	< 0.001
ADAS-Cog	14.0 (4.5)	12.5 (3.6)	− 1.6 (2.4)	< 0.001	15.3 (5.2)	12.4 (4.6)	− 3.0 (3.3)	< 0.001
EVOO (g)	33 (10)	30 (12)	− 3.3 (13)	0.069	36 (12)	26 (6)	− 10 (14)	< 0.001

*MMSE* mini mental state examination, *ADAS-Cog* Alzheimer’s disease assessment scale-cognitive sub-scale, *EVOO* extra virgin olive oil, *Δ* difference

* Paired T test

**Table 5:** Participant’s neuropsychological characteristics after 1 year according to extra virgin olive oil-intake

	MeDiet (N = 55)	MeDiet plus EVOO (N = 55)	*p* value
MMSE	25.6 (1.8)	25.9 (1.3)	0.24
ΔMMSE	0.96 (1.1)	1.3 (1.1)	0.08
ADAS-Cog	12.5 (3.6)	12.4 (4.6)	0.84
ΔADAS-Cog	− 1.6 (2.4)	− 3.0 (3.3)	0.018
ΔADAS-Cog (adjusted^a^)	− 1.6 (0.4)	− 3.0 (0.4)	0.024
ADL	6.0 (0.1)	6.0 (0.1)	0.50
IADL	8.0 (0.1)	8.0 (0.1)	0.32
VF	25 (7)	25 (6)	0.78
BDI-III	11 (6)	12 (6)	0.56

*EVOO* extra virgin olive oil, *MMSE* mini mental state examination, *ADAS-Cog* Alzheimer’s disease assessment scale-cognitive sub-scale, *ADL* activities of daily living, *IADL* instrumental activities of daily living, *VF* verbal fluency, *BDI-III* Beck depression inventory, *Δ* difference

^a^Adjusted mean and standard deviation for alcohol and cheeses (baseline intake) and carbohydrates, fruit and milk (intake after 1 year)

## Discussion

The findings of the present study show a higher reduction of ADAS-cog scores (improved test) after 1 year in the elderly of the MedDiet plus low dose of extravirgin OO group than that observed with a MedDiet alone (− 3.0 ± 0.4 Vs. − 1.6 ± 0.4 respectively; Table 4). This result suggests that, in the short term and despite a low dose, extravirgin OO could be involved in improving the ADAS-cog scores whereas a MedDiet alone slightly preserve cognitive functions. In this study, the two groups had a similar calories and food groups intake at baseline as well as at the second time point (Tables 1, 2, 3), except for alcohol and cheese (at baseline) and carbohydrates, fruit and milk intake (after 1 year). After adjusting the ADAS-cog score variation for these food groups, a higher improvement of ADAS-cog scores was, again, observed in participants of the MedDiet plus low dose of extravirgin OO than in participants at MedDiet alone.

The beneficial effects of the MedDiet on cognitive functions have been attributed to its high MUFA content [6]. However, several evidences raised uncertainty on this association. In the EPIC-Greek cohort, a population of adults aged 60 years old or more evaluated for a period lasting 8 years, MUFA were not associated with MMSE score [11]. A finding from a cross-sectional study reported that an increased consumption of extravirgin OO were independently related to better cognitive functions [33]. However, a specific associations between extravirgin OO intake and cognitive functions or dementia in the short term and with low doses have not been examined yet. Several interventions studies suggest a protective role for OO or extravirgin OO on brain functions over a long period of time. In this regard, one study carried out in a population of community-dwelling individuals at high vascular risk, demonstrated that a MedDiet supplemented with EVOO (1 L/week) was associated with a better global cognitive performance after 6.5 years of follow-up compared with a control low-fat diet [2]. In this study, mean MMSE and Clock Drawing Test (CDT) scores were significantly higher for participants allocated to the MedDiet supplemented with extravirgin OO group in comparison with the control group. However ADAS-cog score was not assessed. In the EPIC—Greek study it has been found that olive oil consumption was weakly positively associated with MMSE score, whereas the association between extravirgin OO and cognition was no tested [11]. Thus, our investigation is original from the point of view of the duration of the study, quality of the OO (only extravirgin) and doses tested. Our results are consistent with that found by Violi et al. [34] Compared with baseline, in healthy subjects of both gender given a Mediterranean-type lunch containing only 10 mg of extravirgin OO, a significant less increase of blood glucose, LDL-Cholesterol and ox-LDL and a more marked increase of blood insulin were detected [34]. It is well accepted that cardiovascular risk factors such as diabetes, dyslipidemia, hypertension and conditions such as insulin-resistance are associated with the development of cognitive impairments [35, 36]. Thus, we assumed that extravirgin OO, even at low dose, may positively affect cognitive functions. Furthermore, in November 2004, the US Federal Drug Administration (FDA) allowed a claim on olive oil labels concerning “the benefits on the risk of coronary heart disease of eating about 23 g of olive oil daily, due to the MUFA in olive oil” [37]. In our study we did find any significant effect on the lipids (data not shown). Nevertheless, since ox-LDL and insulin were not assessed, we cannot rule out these biological effects. In addition, 1 year may be not sufficient to identify cognitive improvements related to reduction of cardiovascular risk factors. We hypothesized that extravirgin OO supplementation rapidly influence cognitive function tests, even at low doses [2, 34–36] whereas the MedDiet may prevent cognitive decline over a long period of time and have a beneficial effect during the long prodromal phase of dementia [38].

EVOO is the best quality oil, produced by mechanically pressing ripe olives, and retains most of its lipophilic components (including alpha-tocopherol, beta-carotene, and phenolic flavonoid compounds) with strong antioxidant properties [39]. In contrast, common olive oil is a mixture of virgin and refined oil, with fewer antioxidant and anti-inflammatory compounds [38]. An industrial solvent extraction of oil from plants involves the majority of the vegetal oils. Thus, while many vegetable oils may contain MUFAs, the industrialized processing of these oils makes them a less desirable choice for health. For these reasons, it is plausible that a low dose (~ 26 g) of extravirgin OO may have different effects on central nervous system from other vegetal oils, as also suggested by some experimental studies. Compared with virgin OO, refined OO is less protective on oxidative damage to lipids, free radical generation and inflammatory activity [40, 41]. Oleuropein is generally the most prominent phenolic compound in olive cultivars, thus, the neuroprotective effects may be mediated by oleuropein [42].

Of course, our study was not designed to investigate which component of OO (oleic acid or phenols) underlie the association with cognitive functions. Since it has been demonstrated that food quality is a key determinant in order to maintain a healthy status [10], our study suggests that a dietary pattern including extravirgin OO, even at low dose, have a protective action on cognitive functions. Several researches have provided evidence of diet-induced changes in cerebral neurotransmitters [43, 44]. Thus, our study may reveal a new approach to prevent the cognitive decline by changing the quality of vegetable oils in the diet.

In this study, as expected, both groups showed an improvement. Our control MedDiet diet improved cognitive functions in line with previous epidemiological and intervention studies [8], possibly through its effect on oxidative stress and inflammation in the brain [32, 39]. However, a higher reduction of ADAS-cog scores was found in the participants of the MedDiet plus extravirgin OO group than that observed with a MedDiet alone (Table 5).

In our population, no indication about calories restriction was given. However, energy intake decreased by about 300 kcal over 1 year. We hypothesize an age-related reduction in energy intake, that is largely a physiologic effect of healthy aging [9]. Furthermore, physical factors such as poor dentition or age-associated changes in taste and smell may limit the type and quantity of food eaten in older people [9].

In this investigation some weaknesses and strengths must be pointed out. It has been suggested that a mean ADAS-Cog change in individuals judged to have clinically relevant change was over three points with an effect size (ES = mean ADAS-cog difference/baseline SD) of more than 0.5 for a minimal clinically relevant change, with values for those not undergoing a clinically significant change being smaller (0.2–0.4) [32]. Thus, in our study in both groups the change seems to be clinically relevant. However, at present, a cut-off point on the ADAS cog that accurately classifies patients in respect of their clinical response is not universally accepted, especially with a dietary intervention. Consequently, caution must be exercised when interpreting our results on the basis of ADAS-cog score change.

To assess how close the food intakes of our population groups were from the Mediterranean Dietary pattern we used MAI, by dividing the sum of the percentage of total energy from typical Mediterranean food groups by the sum of the percentage of total energy from non-typical Mediterranean food groups [30]. The lack of a significant change in the MAI after 1 year was not a surprise since it has been demonstrated that, even though adherence to Mediterranean Diet did not change over time, the consumption of some foods can change toward an healthier food pattern [45]. In addition, our findings are in line with that demonstrated by Fidanza et al. in the elderly [30].

The exclusion of the elderly affected by CVD makes these results not generalizable to a community-dwelling older population but these observations are applicable only to a population with similar characteristics. However, we restricted to individuals without CVD and 1 year of follow-up to reduce the number of potential intermediary events altering the association between the intervention and the studied outcomes. Longer follow-up in the elderly with CVD may be associated with more intermediary events potentially biasing interpretation of the results [46]. In this regard, the rate of drop out during follow-up was of 62% in the MedDiet group. There are no universally agreed criteria for acceptable follow-up rates in nutritional randomized control trials (RCTs) or cohort studies. However, in RCTs, typically investigating drugs, a cut-off of 80% is used in Evidence-Based Medicine (EBM) ‘‘Levels of Evidence’’ to separate ‘‘high’’- and ‘‘low’’-quality randomised trials [47, 48].

They are important points to mention in interpreting the credibility of these findings. Protocol deviations are very common in interventional research. In this study the overall drop-out rate was 53% and it can have implications on the validity and generalizability of research findings. Of course the method of handling data may lead to different conclusions and it is possible that, in this study, the causal relationship cannot be completely established. However, we explored differences in the characteristics of the participants who completed the study versus those who withdrew (Additional file 1: Table S1) and did not differ significantly, suggesting that the drop-out rate did not impact the final results. We performed a common types of sensitivity analyses to assess the robustness of the results in which participants who violate the protocol (missing appointments; discontinued intervention) were excluded from the analysis. Furthermore, while the overall drop-out rate was 53%, it did not affect power because 25 participants each group were required.

Finally, since it is difficult to disentangle OO from the other components of the MedDiet, and aimed to improve adherence to the protocol, we designed this randomized study in which, in the MedDiet plus extravirgin OO group, extravirgin OO was given for free to participants by local oils producers. We believe this is a strength of the study. Further research is needed to establish whether a Mediterranean Diet supplemented with a low dose of extravirgin OO can prevent dementia.

## Conclusion

In this study, we demonstrated that the higher improvement of cognitive functions scores, in a short-term, was observed in individuals of the MedDiet plus low dose (~ 26 g) of extravirgin OO rather than MedDiet alone. In the elderly, a change in the quality of vegetable oils would improve the cognitive functions better than the quantity. Additional studies are required to confirm this results.

## Additional file

**Additional file 1: Table S1.** Baseline participant’s clinical characteristics who completed the study versus those who withdrew.

## Abbreviations

BMI: body mass index
WC: waist circumference
HC: hip circumference
SBP: systolic blood pressure
DBP: diastolic blood pressure
MMSE: mini mental state examination
ADAS-Cog: Alzheimer’s disease assessment scale-cognitive sub-scale
