# Supplementary material for: Effect of the replacement of dietary vegetable oils with a low dose of extravirgin olive oil in the Mediterranean Diet on cognitive functions in the elderly
Source: J Transl Med. 2018 Jan 19;16:10. doi: 10.1186/s12967-018-1386-x (PMC5775590; doi:10.1186/s12967-018-1386-x)
Supplement: Supplementary file 1 — Additional file 1: Table S1. Baseline participant’s clinical characteristics who completed the study versus those who withdrew. [file 12967_2018_1386_MOESM1_ESM.docx]

| Table S1 *Baseline participant’s clinical characteristics who completed the study versus those who withdrew* | | | |
| --- | --- | --- | --- |
| Variables | Participants who  completed the study  (N=110) | Participants who  Withdrew  (n=94) | *P-value* |
| Age (years) | 70(4) | 70(5) | 0.92 |
| Education level (years) | 11(5) | 11(4) | 0.67 |
| MMSE | 24.4(1) | 24.2(2) | 0.72 |
| ADAS-Cog | 15.4(5) | 17.6(9) | 0.085 |
| ADL | 6.0(0.2) | 5.9(0.1) | 0.45 |
| IADL | 7.9(0.5) | 7.8(0.9) | 0.35 |
| VF | 24(7) | 24(9) | 0.83 |
| BDI-III | 12(8) | 14(9) | 0.19 |
| *Prevalence* | | | |
| Obesity (%) | 34 | 39 | 0.84 |
| Hyperlipidemia (%) | 47 | 44 | 0.63 |
| Hypertension (%) | 54 | 60 | 0.70 |
| Diabetes/carbohydrate intolerance (%) | 50 | 55 | 0.34 |

*Note.*MMSE = mini mental state examination; ADAS-Cog = alzheimer’s disease assessment scale-cognitive sub-scale; ADL = activities of daily living;IADL = instrumental activities of dailiy living; VF = verbal fluency;BDI-III = beck depression inventory.
